# Identification of two new flavone 4′-*O*-methyltransferases and their application in *de novo* biosynthesis of (*2S*)-hesperetin in *Yarrowia lipolytica*

**DOI:** 10.1016/j.synbio.2025.03.003

**Published:** 2025-03-20

**Authors:** Yiyun Wang, Ruiqiu Huang, Song Gao, Mingyu Yue, Xuan Zhang, Weizhu Zeng, Bin Tang, Jingwen Zhou, Dongliang Huang, Sha Xu

**Affiliations:** a Engineering Research Center of Ministry of Education on Food Synthetic Biotechnology, and School of Biotechnology, Jiangnan University, 1800 Lihu Road, Wuxi, Jiangsu, 214122, China; b Shenzhen Tianjiao Medical Technology Co., Ltd, GuangDong, Shenzhen, 518029, China; c Science Center for Future Foods, Jiangnan University, 1800 Lihu Road, Wuxi, Jiangsu, 214122, China; d Department of Biomedical Engineering, Southern University of Science and Technology, China

**Keywords:** (2*S*)-Hesperetin, F4′OMT, *Yarrowia lipolytica*, *Citrus reticulata* ‘Chachiensis’, *Citrus grandis* Tomentosa, O-methylated flavonoids

## Abstract

Methyltransferases are pivotal enzymes in the biosynthesis of methylated flavonoids, including (2*S*)-hesperetin. However, existing flavonoid 4′-*O*-methyltransferase (F4′OMT) enzymes typically exhibit low substrate specificity and catalytic efficiency, which hinders microbial synthesis. To overcome this limitation, this study screened and identified two novel F4′OMTs, *Crc*OMT-2 and *Cgt*OMT-3, from Chinese citrus varieties *Citrus reticulata* ‘Chachiensis’ (CZG) and *Citrus grandis* Tomentosa (HZY). These enzymes displayed high substrate specificity for (2*S*)-eriodictyol. A strain capable of *de novo* synthesis of (2*S*)-hesperetin was developed by integrating the novel F4′OMTs and other biosynthetic pathway genes at high copy numbers into *Yarrowia lipolytica*. The engineered strain achieved a remarkable production titre of (2*S*)-hesperetin (130.2 mg/L), surpassing the yields of previously reported F4′OMTs. Furthermore, availability of the cofactor S-adenosylmethionine (SAM) was optimised to enhance methyltransferase catalytic efficiency, enabling the engineered strain to produce 178.2 mg/L of (2*S*)-hesperetin during fed-batch fermentation with SAM supplementation, the highest yield reported to date. This study represents the first successful *de novo* biosynthesis of (2*S*)-hesperetin in *Y. lipolytica*, providing valuable insights into the synthesis of other O-methylated flavonoids.

## Graphical abstract

**Figure:**
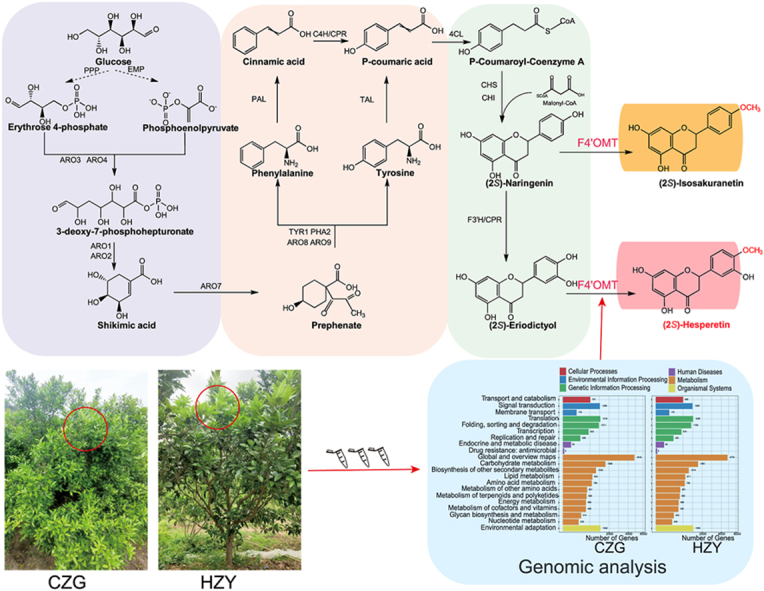

## 1 Introduction

(2*S*)-Hesperetin is a naturally occurring O-methylated flavonoid abundant in fruits, flowers and other parts of various plants [1]. Among them, the content of (2*S*)-hesperidin is relatively high in some citrus plants [2]. It exhibits diverse biological activities including antioxidant, anti-inflammatory, anti-tumour and anti-aging properties, and has significant potential for applications in medicine, dietary supplements and food additives [[3], [4], [5]]. (2*S*)-Hesperetin is mainly produced by chemical synthesis and plant extraction. There are drawbacks for both of these approaches. Plant extraction requires substantial raw plant material and is constrained by seasonal growth and geographic availability [6]. Chemical synthesis entails complex procedures with low yields and relies on toxic reagents and stringent polar reaction conditions [7]. With the development of synthetic biology and analysis of the (2*S*)-hesperetin biosynthesis pathway, microbial biosynthesis is becoming a promising (2*S*)-hesperetin production strategy due to its advantages of easy operation, environmental friendliness, high yield and low cost, providing a sustainable alternative to traditional methods [8,9].

**Fig. 1:**
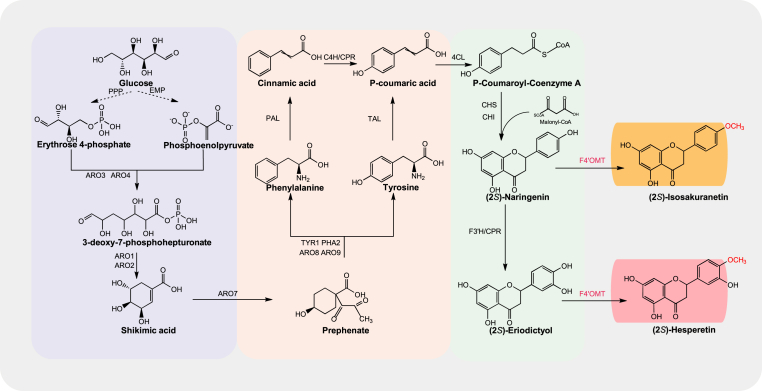
*De novo* (2*S*)-hesperetin biosynthesis pathway in engineered *Y. lipolytica*. PPP, pentose phosphate pathway; EMP, Embden-Meyerhof-Parnas pathway; *ARO3*, 3-deoxy-7-phosphoheptulonate synthase; *ARO4*, 3-deoxy-7-phosphoheptulonate synthase; *ARO1*, pentafunctional AROM polypeptide; *ARO2*, chorismate synthase; *ARO7*, chorismite mutase; *ARO8*, AAA transaminase 1; *ARO9*, AAA transaminase 2; *PAL*, phenylalanine ammonia-lyase; *TAL*, tyrosine ammonia lyase; 4CL, 4-coumarate-CoA ligase; *CHS*, chalcone synthase; *CHI*, chalcone isomerase; *F3H*, flavanone 3-hydroxylase; *F4′OMT*, flavonoid 4′-*O*-methyltransferase.

In plants, the biosynthesis of (2*S*)-naringenin initiates with the transformation of l-phenylalanine or l-tyrosine into *p*-coumaric acid, catalysed by the enzymes phenylalanine ammonia lyase (PAL) and cinnamate 4-hydroxylase (C4H), or alternatively by tyrosine ammonia lyase (TAL). Following this, *p*-coumaric acid is further converted to (2*S*)-naringenin through a series of enzymatic reactions involving 4-hydroxycinnamoyl-CoA ligase (4CL), chalcone synthase (CHS), and chalcone isomerase (CHI). The biosynthetic pathway of (2*S*)-hesperetin originates from (2*S*)-naringenin, which is hydroxylated to (2*S*)-eriodictyol by flavonoid 3′-hydroxylase (F3′H), then methylated to (2*S*)-hesperetin by flavonoid 4′-*O*-methyltransferase (F4′OMT) (Fig. 1) [10]. Previous studies demonstrated the successful heterologous synthesis of (2*S*)-hesperetin in *Escherichia coli* [11]. For example, 14.6 mg/L (2*S*)-hesperetin was produced from 3 mM caffeic acid in *E. coli* 5-alpha [12]. Moreover, various strategies have been employed to enhance (2*S*)-hesperetin production. In one study, 100 mg/L (2*S*)-naringenin was converted to 37.1 mg/L (2*S*)-hesperetin in *E. coli* via whole cell catalysis [10]. In another, catalytic efficiency of F4′OMT was improved through enzyme engineering to produce more (2*S*)-hesperetin [13]. Despite these advances, the titre of *de novo* synthesis remains relatively low. This limitation primarily arises from the weak substrate specificity of F4′OMT [14], which preferentially catalyses the conversion of (2*S*)-naringenin to (2*S*)-isosakuranetin rather than (2*S*)-eriodictyol to (2*S*)-hesperetin, leading to depletion of the precursor (2*S*)-naringenin [15]. Therefore, it is critical to identify or engineer an F4′OMT enzyme with enhanced catalytic specificity for the substrate (2*S*)-eriodictyol.

Methyltransferases (MTs) are a family of enzymes responsible for transferring methyl groups (-CH_3_) from a donor molecule to specific substrates [16]. Based on the different target linkers in substrates, MTs can be classified into four main types: OMTs, NMTs, CMTs and SMTs [17]. F4′OMT utilises S-adenosylmethionine (SAM) as a methyl donor and flavonoids as methyl acceptors, catalysing the transfer of methyl groups from SAM to hydroxyl groups of flavonoids to effect methylation [18]. Consequently, the intracellular methyl supply level is critical for the biosynthesis of (2*S*)-hesperetin. Various strategies have been proposed to improve SAM availability, including enhancing the methionine cycle, overexpressing key enzymes in the SAM biosynthetic pathway, and optimising fermentation conditions [19,20]. Therefore, when designing a (2*S*)-hesperetin cell factory, SAM supply must be carefully considered. *Yarrowia lipolytica* is an important oleaginous yeast with strong acid and salt tolerance, a high-throughput tricarboxylic acid cycle, and sufficient acetyl-CoA precursors [21,22], making it an ideal host for the production of natural products such as flavonoids [23]. The present study aimed to construct the biosynthetic pathway for (2*S*)-hesperetin in *Y. lipolytica* and optimise its synthesis to achieve efficient production.

Firstly, we achieved the efficient synthesis of (2*S*)-hesperetin in *Y. lipolytica* by identifying two new F4′OMTs and optimising the synthesis pathway. We identified two novel F4′OMTs, *Crc*OMT-2 and *Cgt*OMT-3, derived from the Chinese citrus varieties *Citrus reticulata* ‘Chachiensis’ (CZG) and *Citrus grandis Tomentosa* (HZY). These enzymes were integrated into the genome of *Y. lipolytica* YE26, resulting in a strain capable of *de novo* synthesis of (2*S*)-hesperetin. Notably, *Crc*OMT-2 achieved a higher titre of (2*S*)-hesperetin than previously reported F4′OMTs. The optimised strain produced 130.2 mg/L of (2*S*)-hesperetin in fed-batch fermentation, surpassing all previously reported microbial production levels. Furthermore, supplementing the culture with SAM at a final concentration of 1000 mg/L at 16 h enhanced the (2*S*)-hesperetin titre to 178.2 mg/L. This research opens the path for identifying additional F4′OMTs and advancing the synthesis of (2*S*)-hesperetin and its derivatives.

## 2 Materials and methods

### 2.1 Strains and medium

*E. coli* JM109 was used to construct plasmids, while *Y. lipolytica* PO1f served as the primary host strain for synthesising (2*S*)-hesperetin. *E. coli* was cultured at 37 °C on Lysogeny-Broth (LB) medium or agar plates supplemented with ampicillin (100 mg/L). *Y. lipolytica* was cultured on yeast nitrogen base (YNB) medium (6.74 g/L YNB, with or without 0.47 g/L amino acids and 20 g/L glucose) or yeast extract peptone dextrose (YPD) medium (20 g/L glucose, 20 g/L peptone and 10 g/L yeast extract) at 30 °C.

### 2.2 Plasmid construction

Plasmid construction was performed using a Gibson Assembly Kit (Vazyme, Nanjing, China). All heterologous genes were synthesised by Jiangsu Genecefe Biotechnology Co. Ltd. (Wuxi, China). The accuracy of constructs was confirmed by Sanger sequencing (Sangon, Shanghai, China). Construction of *Y. lipolytica* strains and multi-copy integration methods were consistent with previous reports [24]. Transformed yeast cells were streaked on nutrient selective medium and cultured for 3−5 days at 30 °C for subsequent fermentation. Details of all genes, plasmids and strains used in this study are provided in the Supplementary Materials.

### 2.3 Shake flask cultivation and fed-batch fermentation of *Y. lipolytica* strains

Small-scale fermentation was performed in shake flasks. Single colonies were selected from agar plates and cultivated in 5 mL YPD medium for 20−24 h to produce a primary seed solution. A 500 μL volume of primary seed solution was transferred to shake flasks containing 25 mL YPD (2 % v/v) and fermentation was carried out at 30 °C with shaking at 220 rpm for 96 h.

Fed-batch fermentation was conducted in a 5-L bioreactor. Single colonies were selected and cultivated in 10 mL YPD at 30 °C and 220 rpm for 20−24 h to form a primary seed solution. A 10 mL volume of primary seed solution was inoculated into 200 mL YPD for an additional 20−24 h to form a secondary seed solution. The secondary seed solution was added to 2.2 L YPD medium along with 5 mL trace metal solution and 3 mL vitamin solution. The pH was maintained at 5.0 by automatic addition of ammonia water, the cultivation temperature was set at 28 °C, the airflow was 3.0 vvm and the dissolved oxygen content was controlled at 18−22 % through a stirring cascade (300−900 rpm). Once the initial glucose concentration dropped to 2 g/L, 800 g/L D-glucose was added to maintain levels between 0.3 and 1 g/L.

### 2.4 Analytical methods

Optical density at 600 nm (OD_600_) was measured using a SYNERGY H1 microplate reader (BioTek, Palo Alto, CA, USA). For dry cell weight (DCW) measurements, 5 mL of fermentation sample was collected, centrifuged at 12,000 g for 10 min to remove the supernatant, and oven-dried at 60 °C for 60 h to a constant weight. Glucose concentration was measured by centrifuging 1 mL of fermentation sample at 10,000 g for 1 min, filtering the supernatant through a 0.22 μm membrane, and analysing with an S-10 Biosensor Analyzer (Shenzen Sieman Technology Co., Ltd., Shenzhen, China). To detect (2*S*)-hesperetin and other flavonoids, 1 mL of fermentation broth was mixed with 1 mL of methanol, shaken for 10 min, and centrifuged at 13,500 g for 15 min. The upper organic phase was filtered for HPLC analysis using an LC-20AT high-performance liquid chromatography system (Shimadzu, Tianjin, China) equipped with a variable wavelength detector and a ZORBAX Nikpase XDB-C18 column (Agilent, Beijing, China). Detection was performed using a gradient of solvent A (ultrapure water containing 0.1 % trifluoroacetic acid) and solvent B (methanol containing 0.1 % trifluoroacetic acid) at a flow rate of 1 mL/min and a temperature of 40 °C. The gradient was 10–40 % solvent B for 10 min, 40−80 % solvent B for 20 min, 80 % solvent B for 5 min, and 80–10 % solvent B for 5 min. The compounds (2*S*)-naringenin, (2*S*)-eriodictyol, (2*S*)-isosakuranetin and (2*S*)-hesperetin were detected at 350 nm with retention times of 19.9 min, 17.3 min, 25.2 min and 20.9 min, respectively. Quantification was based on calibration curves of the corresponding standards.

The experiment utilized a Liquid Chromatography-Mass Spectrophotometer (LC-MS) from Thermo Electron, Pittsburgh, PA, featuring an electrospray ionization (ESI) source. For the extraction of (2*S*)-hesperetin from the medium, ethyl acetate was employed. The separation and purification of (2*S*)-hesperetin from ethyl acetate were conducted using a BRIX 1860 instrument (Chengda, Beijing, China), which was equipped with a reverse-phase C18 column measuring 25 mm × 70 cm. The mobile phase consisted of 41 % (V/V) methyl alcohol, with a flow rate set at 10 mL/min. Detection was carried out at a wavelength of 290 nm, with an injection volume of 5 mL, and the system was maintained at a constant temperature of 25 °C. Following the chromatographic separation, the collected phase underwent vacuum distillation at 40 °C to remove the organic phase. Subsequently, the aqueous phase was subjected to vacuum freezing to yield (2*S*)-hesperetin in powder form.

### 2.5 Genomic assembly

Leaves of CZG and HZY were collected from Guangdong, China, in October 2022. Genome assembly was initiated using PacBio HiFi sequencing for preliminary genome assembly. Hi-C technology was then applied to obtain relative positional information for genes, allowing for chromosome-level genome assembly [25]. Clean data were searched against Nr, SwissProt, KEGG, TrEMBL, InterPro and GO databases for functional annotation of genes [26]. We have uploaded the original genome sequences to NCBI. The NCBI number for the genome of CZG is PRJNA1173908. The NCBI number for the genome of HZY is PRJNA1173912.

## 3 Results

### 3.1 Exploration and comparison of F4′OMTs

**Fig. 2:**
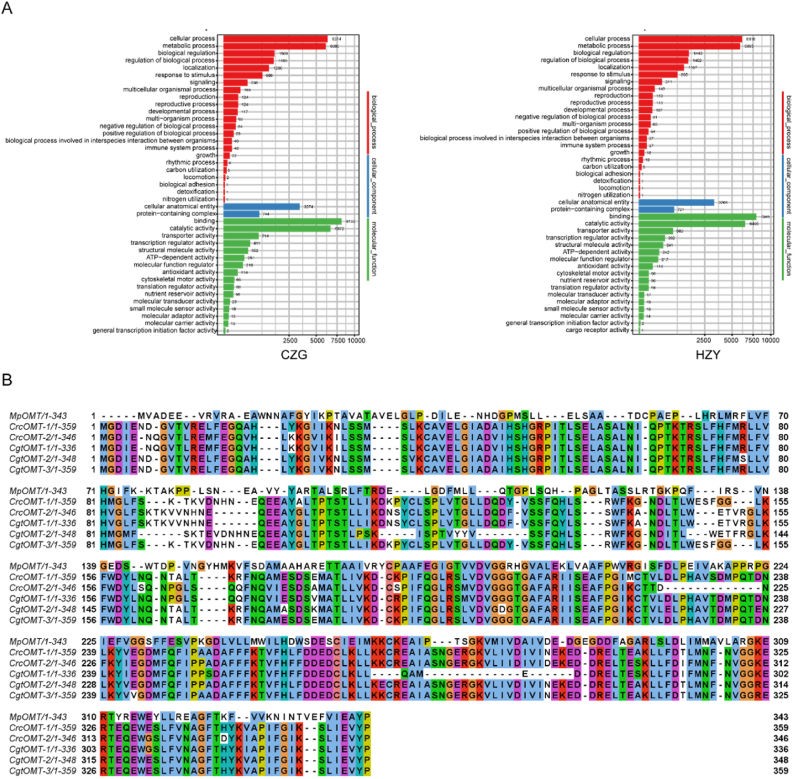
Exploration and comparison of F4′OMTs. (A) Predicted number and functional categorisation of genes identified in the genomes of CZG and HZY. (B) Amino acid sequence alignment comparing the novel F4′OMTs with previously reported MpOMT.

Several functional enzymes associated with flavonoids were identified in the genomes of CZG and HZY. The genome of CZG was predicted to contain 28,855 genes, of which 26,561 were annotated based on comparisons with Nr, SwissProt, KEGG, TrEMBL, InterPro and GO databases. In the HZY genome, 25,991 genes were annotated from 28,956 predicted genes (Fig. 2A). Additionally, several putative MTs were identified in both genomes. Two F4′OMTs, *Crc*OMT-1 and *Crc*OMT-2, were screened from CZG, while three F4′OMTs, *Cgt*OMT-1, *Cgt*OMT-2 and *Cgt*OMT-3, were screened from HZY. Alignments with the *Mp*OMT (Mentha × piperita) amino acid sequence revealed high homology among *Crc*OMT-1, *Crc*OMT-2, *Cgt*OMT-1, *Cgt*OMT-2 and *Cgt*OMT-3, particularly within the central region, which is speculated to contain the enzyme active site (Fig. 2B).

### 3.2 Selection of F4′OMTs

**Fig. 3:**
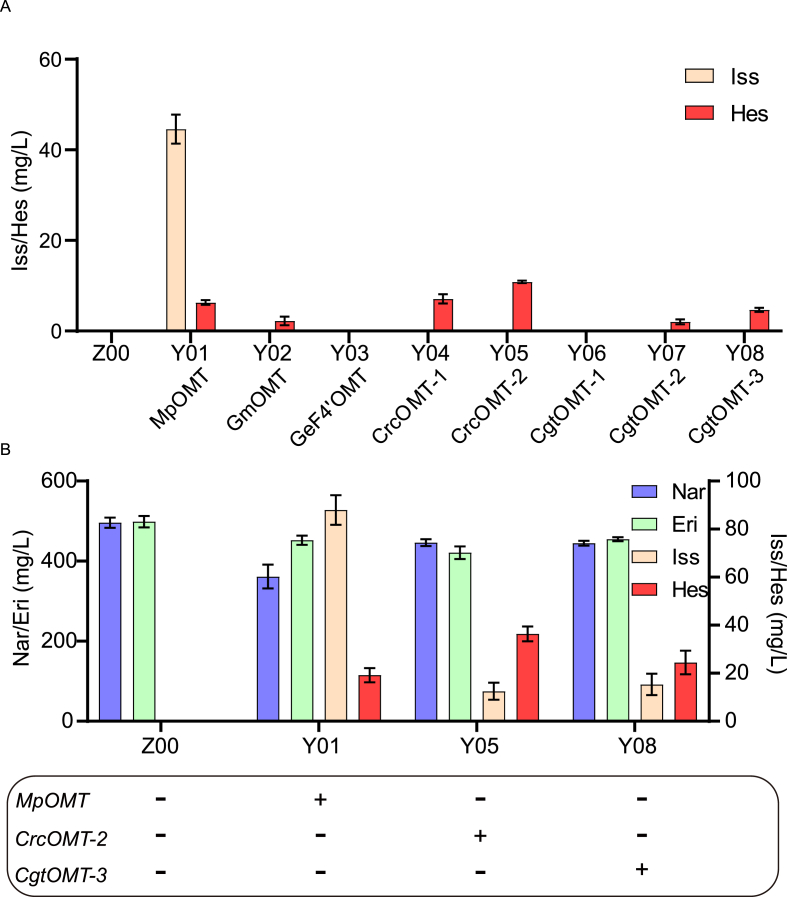
Selection of F4′OMT for biosynthesis of (2*S*)-hesperetin. (A) Eight genes including three reported F4′OMTs (MpOMT, GmOMT and GeF4′OMT), as well as CrcOMT-1, CrcOMT-2, CgtOMT-1, CgtOMT-2 and CgtOMT-3 were overexpressed individually by integration into the genome. (B) Substrate preferences of strains Y01, Y05 and Y08 for (*2S*)-naringenin and (*2S*)-eriodictyol.

F4′OMT exhibits high substrate specificity and catalyses the synthesis of (2*S*)-hesperetin from (2*S*)-eriodictyol and (2*S*)-isosakuranetin from (2*S*)-naringenin [27]. Based on the strain YE26 [23], it will be named Z00 in this article. eight genes, including three reported F4′OMTs, *Mp*OMT, *Gm*OMT (*Glycine max*) and *Ge*F4′OMT (*Glycyrrhiza echinate*), as well as *Crc*OMT-1, *Crc*OMT-2, *Cgt*OMT-1, *Cgt*OMT-2 and *Cgt*OMT-3 were overexpressed individually by integration into the D17 site [28] to form strains Y01–Y08. Liquid chromatography tandem mass spectrometry (LC-MS) analysis revealed that *Mp*OMT, *Gm*OMT, *Crc*OMT-1, *Crc*OMT-2, *Cgt*OMT-2 and *Cgt*OMT-3 all catalysed the production of (2*S*)-hesperetin, while *Mp*OMT, *Crc*OMT-1 and *Cgt*OMT-3 also produced (2*S*)-isosakuranetin (Fig. 3A and Fig. S1). The strain overexpressing *Mp*OMT produced a particularly high titre of (2*S*)-isosakuranetin (Fig. 3A).

Substrate preferences of *Mp*OMT, *Crc*OMT-2 and *Cgt*OMT-3 were further explored. Strains Y01, Y05 and Y08 were cultivated in shake flasks, and a 500 mg/L mixture of (2*S*)-naringenin and (2*S*)-eriodictyol was added at 24 h. After 96 h, flavonoid accumulation was measured in the fermentation broth (Fig. 3B). Strain Y01 predominantly utilized (2*S*)-naringenin to synthesise (2*S*)-isosakuranetin, whereas Y05 and Y08 preferred (2*S*)-eriodictyol to produce (2*S*)-hesperetin. Among the strains, Y05 achieved the highest titre of (2*S*)-hesperetin. Additionally, CrcOMT-2 displayed a higher affinity for (2*S*)-eriodictyol than for (2*S*)-naringenin, although with high (2*S*)-naringenin concentrations it also catalysed the formation of (2*S*)-isosakuranetin. This is contrary to the previous preliminary screening results. The catalytic mechanisms of CrcOMT-2 and CgtOMT-3 are similar to those of MpOMT, but because CrcOMT-2 can synthesise more (2*S*)-hesperitin, it still has differences from the catalytic mechanism of MpOMT. The study of the differences in these catalytic mechanisms requires further analysis from an enzymatic perspective, including the catalytic pockets of the two enzymes.

### 3.3 Construction of a strain for *de novo* synthesis of (2*S*)-hesperetin

**Fig. 4:**
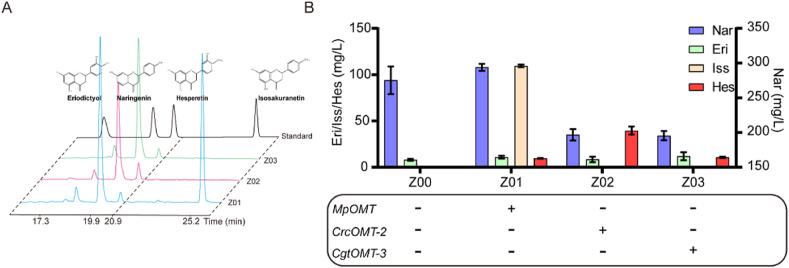
Construction of the *de novo* biosynthetic pathway for (2*S*)-hesperetin. (A) HPCL of four flavonoids and detection of fermentation broth products from strains Z01, Z02 and Z03. The upper organic phase was filtered for HPLC analysis using an LC-20AT high-performance liquid chromatography system equipped with a variable wavelength detector and a ZORBAX Nikpase XDB-C18 column. Detection was performed using a gradient of solvent A (ultrapure water containing 0.1 % trifluoroacetic acid) and solvent B (methanol containing 0.1 % trifluoroacetic acid) at a flow rate of 1 mL/min and a temperature of 40 °C. The gradient was 10–40 % solvent B for 10 min, 40−80 % solvent B for 20 min, 80 % solvent B for 5 min, and 80–10 % solvent B for 5 min. (B) Production of four flavonoids in the fermentation broth of strains Z01, Z02 and Z03 formed following two rounds of multi-copy integration, as well as the control strain Z00. Nar, (*2S*)-naringenin; Eri, (*2S*)-eriodictyol; Iss, (*2S*)-isosakuranetin; Hes, (2*S*)-hesperetin.

A multi-copy genomic integration strategy was employed to construct a *Y. lipolytica* strain capable of *de novo* synthesis of (2*S*)-hesperetin. The chassis strain Z00, already engineered for (2*S*)-eriodictyol synthesis, was used as a starting strain [23]. To enable production of (2*S*)-hesperetin, three key F4′OMT genes, *Mp*OMT, *Crc*OMT-2 and *Cgt*OMT-3, were integrated into the genome of strain Z00 using the 26S rDNA locus with the *URA3* marker. Twenty-four single colonies were randomly selected and cultured in shake flasks for 96 h, with the highest-producing strain chosen for a second round of integration of *Mp*OMT, *Crc*OMT-2 and *Cgt*OMT-3 using ZETA sequences carrying the *LEU* marker. The resulting strains Z01, Z02 and Z03 exhibited distinct production profiles (Fig. 4): Z01 synthesised (2*S*)-hesperetin at 9.7 mg/L along with 109.5 mg/L of (2*S*)-isosakuranetin; Z02 produced the highest titre of (2*S*)-hesperetin at 39.6 mg/L; and Z03 generated (2*S*)-hesperetin with a titre of 10.7 mg/L (Fig. 4).

### 3.4 Enhancement of methyl donors

**Fig. 5:**
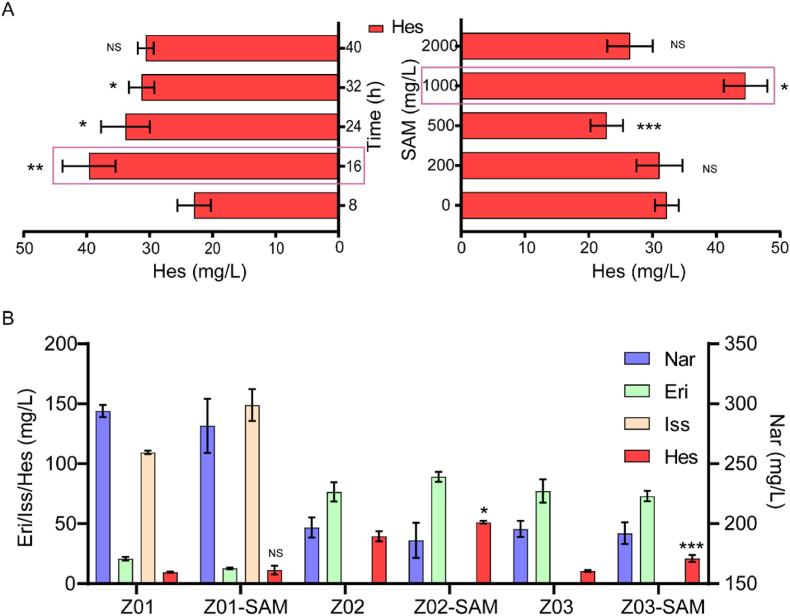
Effect of methyl donor supplementation on (2*S*)-hesperetin production. (A) Optimisation of the time and final concentration of S-adenosylmethionine (SAM) supplementation. (B) Effect of SAM supplementation on the accumulation of four flavonoids in different engineered strains. In these Figures, NS stands for Not Significant, indicating *P* > 0.05; ∗ denotes 0.01 < *P* < 0.05, ∗∗ denotes 0.001 < *P* < 0.01, and ∗∗∗ denotes *P* < 0.001.

Availability of methyl donors is crucial for efficient biosynthesis of (2*S*)-hesperetin, with SAM serving as the primary methyl donor for *O*-MTs in plants [29]. Therefore, the impact of SAM addition timing and concentration on (2*S*)-hesperetin production was evaluated using Z02 as the chassis strain. To determine the optimal timing for SAM supplementation, SAM was added to cultures at a consistent final concentration at various timepoints (8, 16, 24, 32 and 40 h). After 96 h of shake flask fermentation, the highest titre of (2*S*)-hesperetin was achieved by adding SAM at 16 h, indicating this as the optimal time for enhancing methylation efficiency in the biosynthetic pathway. In addition, different final concentrations of SAM (0, 200, 500, 1000 and 2000 mg/L) were added at the optimal 16 h timepoint to observe the effect on (2*S*)-hesperetin yield. The highest (2*S*)-hesperetin production was obtained with a final SAM concentration of 1000 mg/L after 96 h of fermentation (Fig. 5A), suggesting this concentration as optimal for maximising methyl donor availability without causing metabolic stress.

Using the optimised conditions (SAM addition at 16 h at a final concentration of 1000 mg/L), strains Z00, Z01, Z02 and Z03 were fermented for 96 h in shake flasks. The results showed that strain Z01 produced 11.3 mg/L of (2*S*)-hesperetin, a minimal increase, while the titre of (2S)-isosakuranetin was 149 mg/L, a 36 % increase. Strain Z02 produced 51.2 mg/L of (2*S*)-hesperetin, a 29 % increase, and strain Z03 produced 20.1 mg/L, an 88 % increase. Overall, strain Z02 exhibited the strongest ability to produce (2*S*)-hesperetin under the optimised SAM supplementation conditions, highlighting its potential as a robust chassis strain for (2*S*)-hesperetin biosynthesis (Fig. 5B). Given its performance, strain Z02 was selected for further fermentation optimisation.

### 3.5 Fed-batch fermentation

**Fig. 6:**
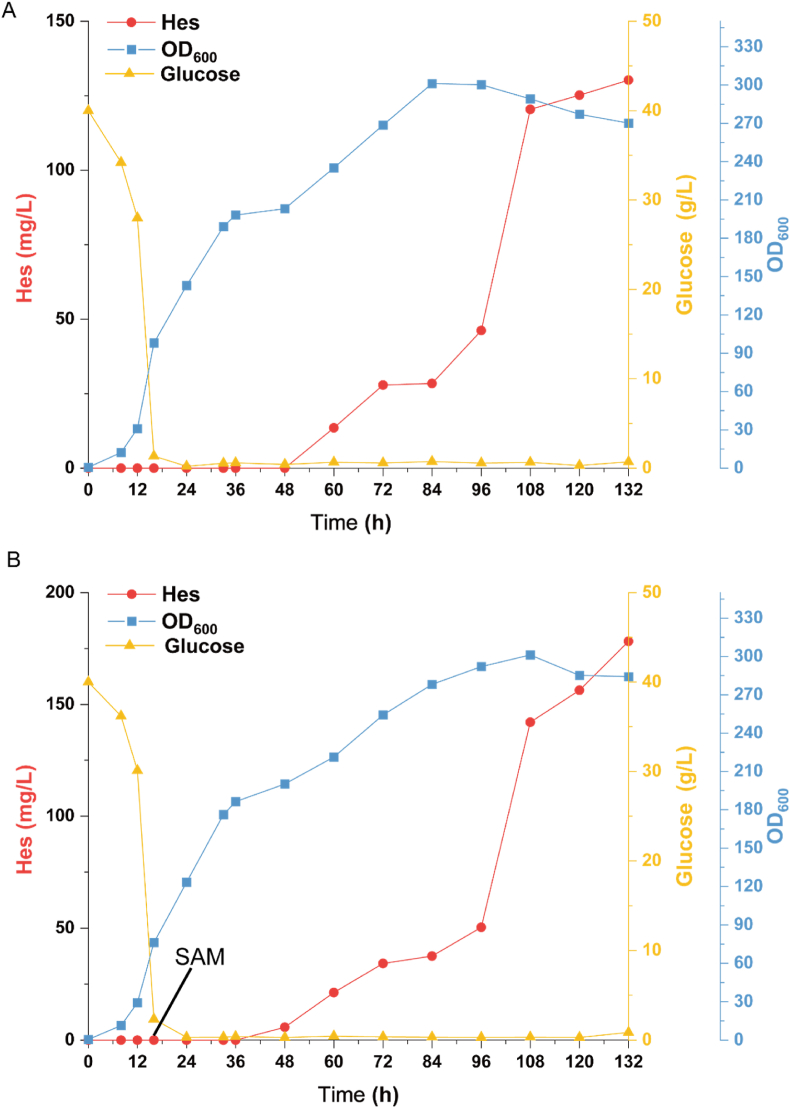
*De novo* biosynthesis of (2*S*)-hesperetin in a 5-L fermenter by strain Z02. (A) Production of (2*S*)-hesperetin with glucose feeding. (B) Production of (2*S*)-hesperetin with glucose feeding and SAM supplementation.

To further evaluate the ability of strain Z02 to produce (2*S*)-hesperetin, fed-batch fermentation was employed in a 5-L bioreactor. Initially, dissolved oxygen was maintained at 18−22 % through aeration and stirring, while pH was controlled at 5.0 by automatic ammonia addition. After 16 h, 40 g of glucose was consumed, leaving only 1.32 g/L remaining in the broth. To maintain a consistent glucose level (0.3−1 g/L), glucose was supplemented at a rate of 30 mL/h, and YPD was added at a rate of 6 mL/h in a 5:1 ratio. The results showed that (2*S*)-hesperetin began accumulating after 48 h, with a significant increase at 96 h, reaching a maximum titre of 130.2 mg/L at 132 h (Fig. 6A). Under the same conditions, addition of SAM (1000 mg/L) at 16 h yielded a maximum (2*S*)-hesperetin titre of 178.2 mg/L at 132 h, a 36.9 % increase (Fig. 6B). This titre is the highest reported to date for microbial production of (2*S*)-hesperetin.

## 4 Discussion

(2*S*)-hesperetin is an O-methylated flavonoid widely used in the food and pharmaceutical industries [30]. *De novo* microbial synthesis of (2*S*)-hesperetin is hindered by the limited substrate specificity of F4′OMTase [31,32]. To overcome this challenge, two novel F4′OMTs, *Crc*OMT-2 and *Cgt*OMT-3, were identified from Chinese citrus cultivars CZG and HZY. *De novo* synthesis of (2*S*)-hesperetin in *Y. lipolytica* was successfully achieved using these newly identified F4′OMTs. *Crc*OMT-2 exhibited a markedly higher (2*S*)-hesperetin production than previously reported F4′OMTs. By supplementing the culture with SAM, the final engineered strain produced 178.2 mg/L of (2*S*)-hesperetin in fed-batch fermentation, achieving the highest yield reported to date for microbial (2*S*)-hesperetin production. Overall, the novel F4′OMTs and strategies adopted in this study hold promise for broad applications for microbial synthesis of (2*S*)-hesperetin and related compounds.

Currently, many methylases from diverse sources, including *Catharanthus roseus* [27], Glycine max (soybean) [33], *Glycyrrhiza uralensis* [34] and mint (Mentha × piperita) [15], have been reported to methylate (2*S*)-eriodictyol to synthesise (2*S*)-hesperetin. However, these methylases exhibit low specificity for (2*S*)-eriodictyol, possessing a broad substrate spectrum and a preference for methylating the precursor (2*S*)-naringenin [9]. For example, Kim et al. (2005) constructed a *de novo* (2*S*)-naringenin synthesis pathway in *E. coli*. Using (2*S*)-naringenin as an intermediate, and after expressing *O*-MT, more (2*S*)-isosakuranetin was generated [35]. Despite adopting various strategies to enhance the substrate specificity of these methylases, the resulting titre of (2*S*)-hesperetin remains low [9]. For instance, *Mp*OMT was incorporated into the *Mp*OMT^S142V^ mutant via directed evolution, which increased the preference for (2*S*)-eriodictyol and resulted in a *de novo* synthesis titre of 27.5 mg/L (2*S*)-hesperetin [15]. By contrast, the present study identified two novel F4′OMTs, *Crc*OMT-2 and *Cgt*OMT-3, through genome sequencing and assembly of CZG and HZY. Although the catalytic efficiency of these F4′OMTs for (2*S*)-eriodictyol remained low, it surpasses that of previously reported enzymes. More importantly, *Crc*OMT-2 and *Cgt*OMT-3 exhibit minimal conversion of (2*S*)-naringenin to (2*S*)-isosakuranetin, demonstrating high substrate specificity for (2*S*)-eriodictyol. However, due to the fact that *Crc*OMT-2 can still catalyze the synthesis of (2*S*)-isosakuranetin from (2*S*)-naringenin, it proves that the problem of substrate heterogeneity of F4′OMTs has not been solved. Future work should focus on using enzyme engineering strategies to explore the similarities and differences between the substrate catalytic pocket of *Crc*OMT-2 and reported F4′OMTs, and to modify the enzyme structure to solve the problem of substrate heterogeneity.

Selection of an appropriate chassis is critical for achieving efficient heterologous synthesis of target products [36]. Numerous flavonoids have been successfully produced in various microorganisms, including the high-yield synthesis of (2*S*)-naringenin in *E. coli* [37], Chrysin and dihydroquercetin in *Saccharomyces cerevisiae* [38,39], and (2*S*)-eriodictyol in *Y. lipolytica* [23]. Currently, heterologous biosynthesis of hesperetin is predominantly conducted in *E. coli* [15]. Despite some progress, production levels remain insufficient to meet industrial demands, likely due to suboptimal expression of plant-derived enzymes in *E. coli* and/or the toxicity of hesperetin and its precursors to the host. *Y. lipolytica*, a widely used non-conventional yeast chassis, offers significant advantages including broad substrate spectrum and high stress tolerance [36,40], making it a promising host for flavonoid biosynthesis [41]. For example, 8.3 g/L (2*S*)-naringenin and 6.8 g/L (2*S*)-eriodictyol have been achieved in a 5-L fermenter using *Y. lipolytica* [23,24]. In the present study, we constructed and optimised the hesperetin biosynthesis pathway in *Y. lipolytica* by integrating multiple gene copies using a multi-copy integration strategy. This approach facilitated the efficient synthesis of (2*S*)-hesperetin, achieving the highest yield reported to date. These results highlight the potential of *Y. lipolytica* as a superior chassis for flavonoid biosynthesis. Although this study has completed the first *de novo* synthesis of (2*S*)-hesperetin in *Y. lipolytica*, the yield has also increased significantly compared to previous reports. But we only performed simple genome multi copy integration of the newly discovered F4′OMT in *Y. lipolytica*. There are still many gene editing modifications that have not been made, such as replacing different promoters, exploring the impact of different copy numbers on final yield, overexpressing genes related to cofactor synthesis to increase the supply of cofactors, and so on.

In the biosynthetic pathways of natural products, the sufficiency of cofactor supply often dictates the efficiency of product synthesis [42]. SAM, the primary methyl donor for most methylation reactions, plays a pivotal role in determining the efficiency of natural product formation [18]. Various strategies have been developed to increase intracellular SAM levels, including designing SAM regeneration systems, supplementing SAM in the fermentation broth, and others [43,44]. For instance, Liu et al. achieved significant improvements in ferulic acid production by developing a methyl supply system driven by betaine [45]. In the present study, we optimised both the timing and concentration of SAM supplementation in the (2*S*)-hesperetin fermentation system, resulting in enhanced methylation efficiency and effective synthesis of (2*S*)-hesperetin. These findings reaffirm that the catalytic activity of F4′OMTs for (2S)-eriodictyol represents the primary bottleneck in (2*S*)-hesperetin biosynthesis. Furthermore, this study underscores the critical importance of ensuring an adequate supply of the SAM cofactor to improve the catalytic performance of F4′OMTs. Moreover, metabolic engineering strategies could be implemented to reconstruct the SAM recycling system, thereby maintaining intracellular SAM homeostasis and further enhancing the efficiency of (2*S*)-hesperetin production. In this study, it was demonstrated that exogenous addition of SAM can indeed increase the production of (2*S*)-hesperetin, but it also incurs certain costs and the increase in (2*S*)-hesperetin production is not significant. In the future, the synthesis pathways of homocysteine and methionine can be strengthened to promote the self accumulation of SAM by engineering bacteria, in order to reduce the additional costs caused by exogenous addition of SAM and further increase the production of (2*S*)-hesperetin.

## 5 Conclusion

In summary, this study discovered two novel F4′OMTs, *Crc*OMT-2 and *Cgt*OMT-3, originating from the Chinese citrus varieties CZG and HZY. These enzymes exhibited high substrate specificity for (2*S*)-eriodictyol and negligible catalytic activity toward the (2*S*)-naringenin to (2*S*)-isosakuranetin side reaction. Additionally, high-copy integration of these F4′OMTs, alongside genes from the (2*S*)-hesperetin biosynthesis pathway, was implemented in *Y. lipolytica*, resulting in an efficient cell factory that achieved a (2*S*)-hesperetin titre of 178.2 mg/L in a 5-L bioreactor with SAM supplementation, the highest yield reported to date. This study provides a valuable reference for the biosynthesis of (2*S*)-hesperetin and other flavonoids, highlighting their potential for industrial-scale production.

## CRediT authorship contribution statement

**Yiyun Wang:** Writing – review & editing, Writing – original draft, Visualization, Investigation. **Ruiqiu Huang:** Writing – review & editing, Writing – original draft, Visualization, Investigation. **Song Gao:** Investigation. **Mingyu Yue:** Investigation. **Xuan Zhang:** Writing – review & editing. **Weizhu Zeng:** Writing – review & editing, Supervision, Resources. **Bin Tang:** Writing – review & editing, Supervision, Resources. **Jingwen Zhou:** Writing – review & editing, Supervision, Funding acquisition. **Dongliang Huang:** Supervision, Funding acquisition. **Sha Xu:** Writing – review & editing, Funding acquisition.

## **Declaration of competing interest**

The authors declare the following financial interests/personal relationships which may be considered as potential competing interests: Ruiqiu Huang and Dongliang Huang are currently employed by Shenzhen Tianjiao Medical Technology Co., Ltd.
