# Supplementary material for: Identification of two new flavone 4′-O-methyltransferases and their application in de novo biosynthesis of (2S)-hesperetin in Yarrowia lipolytica
Source: Synth Syst Biotechnol. 2025 Mar 20;10(3):728–36. doi: 10.1016/j.synbio.2025.03.003 (PMC12002713; doi:10.1016/j.synbio.2025.03.003)
Supplement: Multimedia component 1 [file mmc1.docx]

**Supplementary Materials**

**Identification of two new flavone 4'-*O*-methyltransferases and their application in *de novo* biosynthesis of (*2S*)-hesperetin in *Yarrowia lipolytica***

Yiyun Wang^1,#^, Ruiqiu Huang^2,#^, Song Gao^1^, Mingyu Yue^1^, Xuan Zhang^1^,Weizhu Zeng^1,3^, Bin Tang^4^, Jingwen Zhou^1,3^, Dongliang Huang^2,*^, Sha Xu^1*^

^1^Engineering Research Center of Ministry of Education on Food Synthetic Biotechnology, and School of Biotechnology, Jiangnan University, 1800 Lihu Road, Wuxi, Jiangsu 214122, China;

^2^Shenzhen Tianjiao Medical Technology Co., Ltd, Shenzhen，518029, GuangDong,

^3^Science Center for Future Foods, Jiangnan University, 1800 Lihu Road, Wuxi, Jiangsu 214122, China;

^4^Department of Biomedical Engineering, Southern University of Science and Technology

* Correspondence to:

Dongliang Huang, Shenzhen Tianjiao Medical Technology Co., Ltd, Shenzhen，518029, GuangDong, China.

E-mail: huangdl88@aliyun.com

Sha Xu, School of Biotechnology, Jiangnan University, 1800 Lihu Rd, Wuxi, Jiangsu 214122, China.

Phone: +86-510-85914371, Fax: +86-510-85914371

E-mail: xusha1984@jiangnan.edu.cn

**Supplementary Tables**

**Table S1. Strains used in this study.**

| **Strains** | **Relevant properties or genotype** | **Sources** |
| --- | --- | --- |
| *E. coli* | JM109 | Sangon Biotech |
| Po1f | *Y. lipolytica* W29, *MATa*, *URA3-302, LEU2-270, XPR2-322, AXP-2* | Madzak et al.[1] |
| Z00 | YE26[2] | Our laboratory |
| Y01 | Z00, D17::*P_TEF_-MpOMT -T_XPR2_* | This study |
| Y01 | Z00, D17::*P_TEF_-MpOMT -T_XPR2_* | This study |
| Y02 | Z00, D17::*P_TEF_-GmOMT -T_XPR2_* | This study |
| Y03 | Z00, D17::*P_TEF_-* *GeF4'OMT -T_XPR2_* | This study |
| Y04 | Z00, D17::*P_TEF_-* *CrcOMT-1 -T_XPR2_* | This study |
| Y05 | Z00, D17::*P_TEF_-* *CrcOMT-2 -T_XPR2_* | This study |
| Y06 | Z00, D17::*P_TEF_-* *CgtOMT-1 -T_XPR2_* | This study |
| Y07 | Z00, D17::*P_TEF_-* *CgtOMT-2 -T_XPR2_* | This study |
| Y08 | Z00, D17::*P_TEF_-* *CgtOMT-3 -T_XPR2_* | This study |
| Z01 | Z00, rDNA::*P_TEF_-MpOMT-T_XPR2_*_;_ ZETA::*P_TEF_-MpOMT-T_XPR2_* | This study |
| Z02 | Z00, rDNA::*P_TEF_-CrcOMT-2-T_XPR2_*_;_ ZETA::*P_TEF_- CrcOMT-2 -T_XPR2_* | This study |
| Z03 | Z00, rDNA::*P_TEF_-CgtOMT-3-T_XPR2_*_;_ ZETA::*P_TEF_- CgtOMT-3 -T_XPR2_* | This study |

**Table S2. Plasmids used in this study**

| **Plasmid** | **Description** | **Source** |
| --- | --- | --- |
| pYLXP' | AMP, *LEU2* marker, TEF promoter and XPR2 terminator | Our laboratory |
| pYLXP'2 | AMP, *URA3* marker, TEF promoter and XPR2 terminator | Our laboratory |
| PYlY01 | pYLXP', D17-*P_TEF_- MpOMT -T_XPR2_* | This study |
| PYlY02 | pYLXP', D17-*P_TEF_- GmOMT -T_XPR2_* | This study |
| PYlY03 | pYLXP', D17-*P_TEF_- GeF4'OMT -T_XPR2_* | This study |
| PYlY04 | pYLXP', D17-*P_TEF_- CrcOMT-1 -T_XPR2_* | This study |
| PYlY05 | pYLXP', D17-*P_TEF_- CrcOMT-2 -T_XPR2_* | This study |
| PYlY06 | pYLXP', D17-*P_TEF_- CgtOMT-1 -T_XPR2_* | This study |
| PYlY07 | pYLXP', D17-*P_TEF_- CgtOMT-2 -T_XPR2_* | This study |
| PYlY08 | pYLXP', D17-*P_TEF_- CgtOMT-3 -T_XPR2_* | This study |
| PYlZ01 | pYLXP’, rDNA-*P_TEF_- MpOMT -T_XPR2-_ P_pdh_- LEU2-T_XPR2_* | This study |
| PYlZ02 | pYLXP’, rDNA-*P_TEF_- CrcOMT-2 -T_XPR2-_ P_pdh_- LEU2-T_XPR2_* | This study |
| PYlZ03 | pYLXP’, rDNA-*P_TEF_- CgtOMT-3 -T_XPR2-_ P_pdh_- LEU2-T_XPR2_* | This study |
| PYlR01 | pYLXP’2, ZETA-*P_TEF_- MpOMT -T_XPR2-_ P_pdh_- URA3-T_XPR2_* | This study |
| PYlR02 | pYLXP’2, ZETA-*P_TEF_- CrcOMT-2 -T_XPR2-_ P_pdh_- URA3-T_XPR2_* | This study |
| PYlR03 | pYLXP’2, ZETA-*P_TEF_- CgtOMT-3 -T_XPR2-_ P_pdh_- URA3-T_XPR2_* | This study |

**Table S3. Main primers and synthetic oligos used in this study**

| **Primers** | **Nucleotide sequence (5'-3')** | **Description** |
| --- | --- | --- |
| F1 | GTTGCTGACGAAGAAGTCAG | Forward primer for amplification of  MpOMT |
| R1 | TCATGGGTATGCTTCAATGACAAATTCAA | Reverse primer for amplification of  MpOMT |
| F2 | GTTGAACTGGATATCCCGG | Forward primer for amplification of  GmOMT |
| R2 | TTACGGGTAGATTTCGATCAGGCTCAG | Reverse primer for amplification of  GmOMT |
| F3 | GCTTTTTCTACTAATGGTAG | Forward primer for amplification of  GeF4'OMT |
| R3 | CAAAGATTTAAAACCACAAATTG | Reverse primer for amplification of  GeF4'OMT |
| F4 | GGTGACATTGAGAACGACGGTGTG | Forward primer for amplification of  CrcOMT-1 |
| R4 | GGGTAGACCTCGATCAGAGAC | Reverse primer for amplification of  CrcOMT-1 |
| F5 | GGTGACATTGAGAACCAGGGC | Forward primer for amplification of  CrcOMT-2 |
| R5 | TTAGGGGTACACCTCGATCAGAG | Reverse primer for amplification of  CrcOMT-2 |
| F6 | GGTGACATTGAGAACCAGGGCGTGAC | Forward primer for amplification of  CgtOMT-1 |
| R6 | TAGGGGTAGACCTCGATCAGGGAC | Reverse primer for amplification of  CgtOMT-1 |
| F7 | GGTGACATCGAGAACGACGGTG | Forward primer for amplification of  CgtOMT-2 |
| R7 | TTAGGGGTACACCTCGATCAGGG | Reverse primer for amplification of  CgtOMT-2 |
| F8 | GCAGCAGTGTCTGTAGCAGTGT | Forward primer for amplification of  CgtOMT-3 |
| R8 | CTGCCATCGAGAACGACGGTGTAC | Reverse primer for amplification of  CgtOMT-3 |
|  |  |  |

**Table S4. The sequences of some genes used in this study**

|  | **Sequence (5'-3')** |
| --- | --- |
| **MpOMT** | ATGGTTGCTGACGAAGAAGTCAGAGTTAGAGCTGAAGCCTGGAACAACGCTTTCGGTTACATCAAGCCAACTGCCGTTGCTACCGCCGTGGAACTAGGGTTACCAGACATCTTGGAAAACCACGACGGCCCAATGTCCCTGTTGGAATTATCTGCTGCTACTGACTGCCCAGCTGAACCATTGCACAGATTGATGAGATTCTTGGTTTTCCACGGTATCTTCAAGAAGACCGCTAAGCCTCCATTGTCTAACGAAGCTGTCTACTACGCCCGTACAGCTTTGTCCAGATTGTTTACCAGAGACGAGTTGGGTGATTTCATGTTATTGCAAACTGGTCCATTGTCCCAACACCCGGCCGGTTTGACTGCTTCCTCACTAAGAACCGGTAAACCTCAATTCATCAGATCTGTTAACGGTGAAGACTCTTGGACTGACCCAGTCAATGGTTATCACATGAAGGTCTTCTCCGACGCTATGGCTGCTCACGCACGTGAAACCACTGCCGCCATCGTTAGATACTGTCCAGCTGCCTTTGAAGGTATCGGTACTGTTGTCGATGTTGGTGGTAGACACGGTGTCGCTTTGGAAAAGTTGGTCGCCGCTTTCCCTTGGGTTCGTGGTATCTCTTTCGATTTGCCAGAAATTGTCGCTAAGGCTCCACCAAGACCAGGTATTGAATTTGTTGGTGGTTCTTTCTTCGAAAGTGTTCCAAAAGGTGACTTAGTTTTGTTGATGTGGATTTTACATGACTGGTCTGATGAATCTTGTATCGAAATCATGAAAAAGTGTAAGGAAGCCATTCCAACCTCCGGTAAGGTTATGATTGTCGATGCTATTGTTGATGAAGATGGTGAAGGTGATGATTTCGCTGGTGCTAGATTATCTTTGGACTTGATCATGATGGCTGTCTTGGCTAGAGGTAAGGAAAGAACTTACAGAGAATGGGAATACTTGTTGAGAGAAGCTGGTTTCACCAAGTTCGTTGTTAAGAACATTAACACTGTTGAATTTGTCATTGAAGCATACCCATGA |
| **GmOMT** | ATGGTTGAACTGGATATCCCGGATATCATCCAGAGCGATTCTCACGGTCAGCCGATCACCTTCTCTGAACTGGTTAGCATCCTGCAGGTTCCGCCGACCAAAACCCGTCAGGTTCAGAGCCTGATGCGTTACCTGGCGCACAACGGCTTCTTCGAAATCGTTCGTATCCACGATAACATCGAAGCGTACGCTCTGACCGCGGCGTCTGAACTGCTGGTTAAAAGCAGCGAACTGAGCCTGGCGCCGATGGTTGAATACTTCCTGGAACCGAACTGCCAGGGCGCGTGGAACCAGCTGAAACGTTGGGTTCACGAAGAAGATCTGACCGTTTTCGAAGTTAGCCTGGGCACCCCGTTCTGGGATTTCATCAACAAAGATCCGGCGTACAACAAATCTTTCAACGAAGCGATGGCGTGTGATAGCCAGATGCTGAACCTGGCTTTCCGTGATTGCAACTGGGTTTTCGAAGGCCTGGAAAGCATCGTTGATGTTGGCGGCGGCACCGGCATCACCGCGAAAATCATCTGCGAAGCGTTCCCGAAACTGAAATGCATGGTTCTGGAACGTCCGAACGTTGTTGAAAACCTGAGCGGCTCTAACAACCTGACCTTCGTTGGTGGTGATATGTTCAAATGCATCCCGAAAGCGGATGCGGTTCTGCTGAAACTGGTGCTGCACAACTGGAACGATAACGACTGCATGAAAATCCTGGAAAACTGCAAAGAAGCGATCAGCGGCGAATCCAAAACCGGTAAAGTTGTTGTTATCGATACCGTTATCAACGAAAACAAAGATGAACGTCAGGTTACCGAACTGAAACTGCTGATGGACGTGCACATGGCGTGCATCATCAACGGTAAAGAACGTAAAGAAGAAGATTGGAAAAAACTGTTCATGGAAGCGGGTTTCCAGAGCTACAAAATTAGCCCGTTCACCGGTTACCTGAGCCTGATCGAAATCTACCCGTAA |
| **GeF4'OMT** | ATGGCTTTTTCTACTAATGGTAGTGAAGAAATTGAATTATATCATGCACAAATTCATTTGTATAAACATGTTTATAATTTTGTTTCTTCAATGGCATTGAAATCAGCAATGGAATTAGGTATAGCTGATGTAATTCATAATCATGGTAAACCTATTACATTGCCTGAATTAGCATCTGCATTGAAATTACATCCATCTAAAGTAGGTATTTTATATAGATTTTTAAGATTGTTAACTCATAATGGTTTCTTTGCTAAAACTACTGTTCCATCACAAAATGGTAAAGATGGTGAAGAAGAAGAAGAAACCGCTTATGCATTGACTCCACCATCTAAATTGTTGGTTAAAGGTAAACCAACATGTTTAGCTTCTATTGTTAGAGGTGCTTTGCACCCATCTAGTTTAGATATGTGGAGATCTTCTGAAAAATGGTTTAAAGAAGATAAAGAATTGACTTTATTTGAATCAGCTACAGGTGAATCTTTTTGGGATTTCTTGAACAAAGATTCTGAATCAGGTACATTATCTATGTTTCAAGAAGCTATGGCTGCTGATTCACAAATGTTTAAATTAGCTTTAAAAGAATGTAGACATGTTTTTGAAGGTTTAGAATCTTTGGTTGATGTTGGTGGTGGTACTGGTGGTGTTACTAAATTAATTCATGAAGAATTTCCACATTTGAAATGTACTGTTTTTGATCAACCACAAGTTGTTGGTAACTTGTCAGGTAATGAAAACTTAAAATTTGTCGGTGGTGACATGTTTAAATCTATACCTCCAGCTGATGCTGTTTTGTTAAAATGGGTTTTGCATGATTGGAATGATGAATTATCTTTAAAAATTTTAAAAAATTCTAAAGAAGCTATTTCTGGTAAAGGTAAAGAAGGTAAAGTTATAATTATAGATATATCAATTGATGAAGCTTCTGGTGACAGAGAATTAACTGAATTACAATTAGATTATGACTTAGTTATGTTAACCATGTTTAATGGTAAAGAAAGAGAAAAGAAAGAATGGGAAAAATTGATTAGTGATGCTGGTTTTTCTTCTTATAAAATTACTCCAATTTGTGGTTTTAAATCTTTGATTGAAGTTTTTCCATAA |
| **CrcOMT-1** | ATGGGTGACATTGAGAACGACGGTGTGACCGTGCGAGAGCTGTTCGAGGGACAGGCTCACCTGTACAAGGGCATCATCAAGAACCTGTCTTCCATGTCTCTGAAGTGTGCCGTCGAGCTGGGTATCGCCGACGCTATTCACTCCCACGGCCGACCTATCACCCTGTCTGAGCTGGCTTCTGCCCTGAACATCCAGCCCACCAAGACCCGATCCCTGTTCCACTTCATGCGACTGCTGGTGCACATGGGTCTGTTCTCCAAGACCAAGGTCGACAACCACAACGAGCAGGAGGAGGCCTACGCCCTTACCCCTACTTCTACCCTGCTGATCAAGGACAAGCCCTACTGTCTGTCCCCCCTGGTGACCGGTCTGCTTGATCAGGACTACGTCTCCTCCTTCCAGCACCTGTCCCGATGGTTCAAGGGCAACGACCTGACCCTGTGGGAGTCTTTCGGCGGTCTGAAGTTCTGGGACTACCTGAACCAGAACACCGCCCTGACCAAGCGATTCAACCAGGCTATGGAATCTGACTCTGAGATGGCCACCCTGATCGTGAAGGACTGCAAGCCCATCTTCCAGGGTCTGCGATCCCTGGTGGACGTCGGTGGTGGTACTGGTGCTTTCGCCCGAATCATTTCCGAGGCCTTCCCCGGAATTATGTGTACCGTGCTGGACCTGCCCCACGCTGTTTCTGACATGCCCCAGACCGACAACCTGAAGTACGTGGAGGGCGACATGTTCCAGTTCATCCCCGCCGCTGACGCCTTCTTCTTCAAGACCGTGTTCCACCTGTTCGACGACGAGGACTGTCTGAAGCTGCTGAAGAAGTGCCGAGAGGCCATTGCCTCCAACGGCGAGCGAGGTAAAGTCCTGATCGTCGACATTGTGATCAACGAGAAGGAGGACGACCGAGAGCTGACCGAGGCTAAGCTGCTGTTCGACACCCTGATGAACTTCAACGTCGGTGGTAAAGAGCGAACCGAGCAGGAGTGGGAGTCTCTGTTCGTCAACGCCGGTTTCACCCACTACAAGGTCGCCCCTATCTTCGGTATCAAGTCTCTGATCGAGGTCTACCCCTAA |
| **CrcOMT-2** | ATGGGTGACATTGAGAACCAGGGCGTCACCCTGCGAGAGATGTTCGAGGGACAGGTGCACCTGAAGAAGGGTGTGATCAAGATCCTGTCCTCCATGTCTCTGAAGTGCGCCGTGGAGCTGGGCATCGCTGATGTTATCCACTCCCACGGTCGACCCATCACCCTGTCTGAGCTGGCTTCCGCTCTGAACATTCAGCCCACCAAGACCCGATCTCTGTTCCACTTCATGCGACTGTTCGTCCACGTGGGTCTGTTCTCTAAGACCAAGGTGGTGAACCACAACGAGCAGGAGGAGGCCTACGGCCTTACCCCTACTTCTACCCTGCTGATTAAGGACAACTCCTACTGCCTGTCCCCCCTGGTCACCGGTCTTCTTGACCAGGACTTCGTGTCCTCTTTCCAGTACCTGTCTTCTTGGTTCAAGGCCAACGACCTGACCCTGTGGGAGACCGTTCGAGGTCTGAAGTTCTGGGACTACCTGTCCCAGAACCCCGGCCTTTCCCAGCAGTTCAACCAGGTCATTGAGTCTGACTCCGCTATGGCTACCCTGCTGGTGAAGGACTGTTCCCCCATCTTCCAGGGTCTGCGATCTATGGTGGACGTCGGTGGTGGCACCGGAGCTTTTGCTCGAATTATCTCTGAGGCCTTCCCCGGCATTAAGTGTACCACCGACAACTTCAAGTACATTGAGGGTGACATGTTCCAGTTCATTCCCCCCGCCGACGCCTTCTTCTTCAAGACTGTCTTCCACTTCTTCGACGACGAGGACTGTCTGAAGCTGCTGAAGAAGTGTCGAGAGGCCATCGCCTCTAACGGTGAGCGAGGTAAAGTCCTGATCATTGACATTGTGATTGACGAGAAGGAGGACGACCGAGAGCTGACCGAGTCTAAGCTGCTGTTCGACATCTTCATGAACTTCAACGTGGGTGGTAAAGAGCGAACCGAGCAGGAGTGGGGCTCCCTGTTTGTGAACGCCGGTTTCACCGACTACAAGATCGCCCCCATCTTCGGCATTAAGTCTCTGATCGAGGTGTACCCCTAA |
| **CgtOMT-1** | ATGGGTGACATTGAGAACCAGGGCGTGACCCTGCGAGAGATGTTCGAGGGACAGGTCCACCTGAAGAAGGGTGTCATCAAGATCCTGTCTTCTATGTCTCTGAAGTGCGCCGTCGAGCTGGGCATCGCTGATGTTATTCACTCCCACGGTCGACCCATCACCCTGTCTGAGCTGGCTTCTGCCCTGAACATTCAGCCCACCAAGACCCGATCCCTGTTCCACTTCATGCGACTGTTCGTCCACGTGGGCCTGTTCTCCAAGACCAAGGTCGTGAACCACAACGAGCAGGAGGAGGCCTACGGCCTTACCCCTACTTCCACCCTGCTGATTAAGGACAACTCTTACTGCCTGTCCCCCCTGGTCACCGGACTTCTTGACCAGGACTTCGTCTCCTCCTTCCAGTACCTGTCCTCCTGGTTCAAGGCCAACGACCTGACCCTGTGGGAGACCGTTCGAGGTCTGAAGTTCTGGGACTACCTGAACCAGAACCCCGGCCTGTCTCAGCAGTTCAACCAGGTCATTGAGTCTGACTCCGTGATGGCCACCCTGCTGGTCAAGGACTGTCGACCTATTTTCCAGGGTCTGCGATCTATGGTTGACGTCGGTGGCGGTACCGGCGCTTTTGCTCGAATTATCTCTGAGGCCTTCCCCGGTATCAAGTGTACCGTGCTGGACCTGCCCCACGCTGTTACTGACATGCCCCAGACCGACAACCTGAAGTACATCGAGGGCGACATGTTCCAGTTCATCCCCCCCTCCGACGCCTTCTTCTTCAAGACCGTCTTCCACTTCTTCGACGACGAGGACTGCCTGAAGCTGCTGCAGGCTATGGAGGACGACCGAGAGCTGACCGAGTCTAAGCTGCTGTTCGACATCTTCATGAACTTCAACGTGGGCGGCAAGGAGCGAACCGAGCAGGAGTGGGGTTCTCTGTTCGTCAACGCCGGTTTCACCCACTACAAGATCGCCCCCATCTTCGGCATTAAGTCCCTGATCGAGGTCTACCCCTAA |
| **CgtOMT-2** | ATGGGTGACATCGAGAACGACGGTGTGACCGTGCGAGAGCTGTTCGAGGGACAGGCTCACCTGTACAAGGGCATCGTCAAGAACCTGTCCTCCATGTCTGTGAAGTGCGCCGTGGAGCTGGGCATTGCTGACGCTATCCACTCCCACGGCCGACCTATTACCCTGTCTGAGCTGGCCTCCGCCCTGAACATTCAGCCTACCAAGACCCGATCCCTGTTCCACTTCATGTCTCTGCTGGTGCACATGGGCATGTTCTCCAAGACCGAGGTCGACAACCACAACGAGCAGGAGGAGGCCTACGGCCTTACCCCTACTTCTACCCTGCCCTCCAAGATCTCCCCCACCGTTTACTACGTGTCTTCCTTCCAGCACCTGTCCCGATGGTTCAAGGGCAACGACATTACCCTGTGGGAGACCTTCCGAGGCCTGAAGTTCTGGGACTACCTGAACCAGAACACCGCCCTGACCAAGCGATTCAACCAGGCTATGGCTTCTGACTCCAAGATGGCCACCCTGATTGTCAAGGACTGTAAGCCCATCTTCCAGGGCCTGCGATCTCTGGTGGACGTGGGTGACGGAACCGGAGCTTTCGCTCGAATCATTTCTGAGGCCTTCCCCGGCATCAAGTGCACCGTTCTGGAGCTGCCCCACGCTGTTACCGACATGCCTCAGACCGAGAACCTGAAGTACGTCGAGGGCGACATGTTCCAGTTCATTCCCCCCGCCGACGCCTTCTTCTTCAAGACTGTGTTCCACCTGTTCGACGACGAGGACTGTCTGAAGCTGCTGAAGGAGTGCCGAGAGGCCATCGCTTCCAACGGAGAGCGAGGTAAAGTGCTGATCGTGGACATCGTCATCAACGAGAAGGAGGACGACCGAGAGCTGACCGAGGCTAAGCTGCTGTTCGACACCCTGATGAACTTCAACGTCGGTGGTCGAGAGCGAACCGAGCAGGAGTGGGAGTCTCTGTTCGTGAACGCCGGTTTCACCCACTACAAGGTCGCCCCTATTTTCGGCATCAAGTCCCTGATCGAGGTGTACCCCTAA |
| **CgtOMT-3** | ATGGGTGACATCGAGAACGACGGTGTGACCGTGCGAGAGCTGTTCGAGGGACAGGCTCACCTGTACAAGGGCATCGTCAAGAACCTGTCTTCCATGTCTCTGAAGTGCGCCGTGGAGCTGGGTATTGCCGACGCTATTCACTCTCACGGTCGACCCATCACCCTGTCTGAGCTGGCTTCTGCCCTGAACATCCAGCCCACCAAGACCCGATCCCTGTTCCACTTCATGCGACTGCTGGTCCACATGGGTCTGTTCTCCAAGACCAAGGTGGACAACCACAACGAGCAGGAGGAGGCCTACGGCCTTACCCCTACTTCTACCCTGCTGATTAAGGACAAGCCCTACTGCCTGTCCCCCCTGGTTACCGGTCTGCTTGACCAGGACTACGTCTCTTCCTTCCAGCACCTGTCTCGATGGTTCAAGGGTAACGACCTGACCCTGTGGGAGTCTTTCGGTGGTCTGAAGTTCTGGGACTACCTGAACCAGAACACCGCCCTGACCAAGCGATTCAACCAGGCTATGGAATCTGACTCTGAGATGGCCACCCTGATCGTCAAGGACTGCAAGCCCATTTTCCAGGGTCTGCGATCTCTGGTCGACGTGGGTGGAGGAACCGGAGCTTTCGCTCGAATTATTTCTGAGGCCTTCCCCGGTATCAAGTGCACCGTGCTGGACCTGCCCCACGCTGTCACCGACATGCCTCAGACTGACAACCTGAAGTACGTCGTGGGTGACATGTTCCAGTTCATTTGCACCGCCGACGCCTTCTTCTTCAAGACTGTGTTCCACCTGTTCGACGACGAGGACTGCCTGAAGCTGCTGAAGAAGTGCCGAGAGGCCATCGCCTCCAACGGAGAGCGAGGTAAAGTGCTGATCGTGGACATCGTGATCAACGAGAAGGAGGACGACCGAGAGCTGACCGAGGCTGAGCTGCTGTTCGACACCCTGATGAACTTCAACGTCGGCGGCCGAGAGCGAACCGAGCAGGAGTGGGAGTCTCTGTTCGTGAACGCCGGAGCCACCCACTACAAGGTCGCCCCTATTTTCGGTATTAAGTCTCTGATCGAGGTCTGCCTATAA |


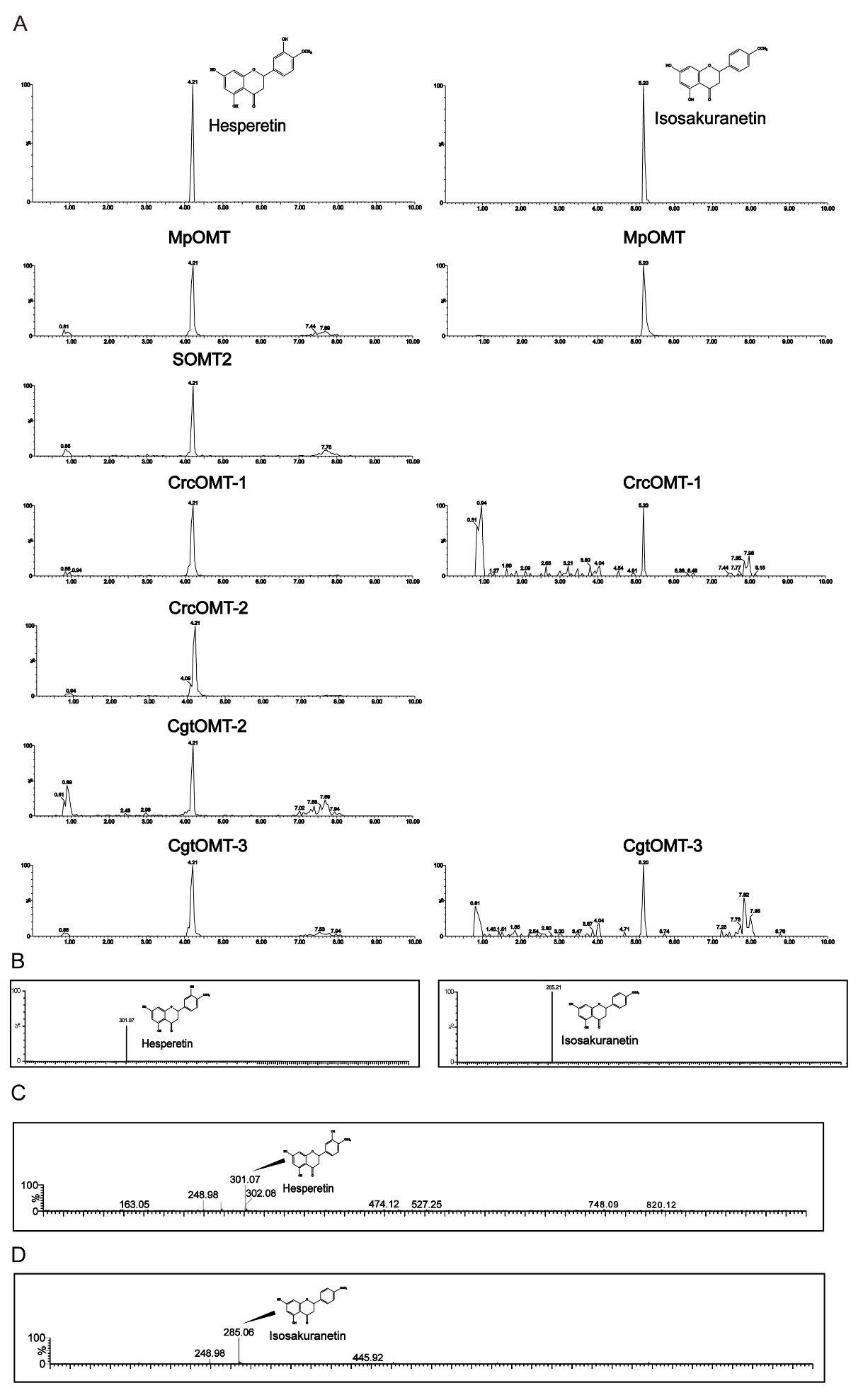


**Figure.S1 Results of liquid phase and ultra-high performance liquid chromatography tandem quadrupole time-of-flight mass spectrometry detection.**

(A) Phase diagram of the production solution of (2*S*)-hesperetin and (2*S*)-isosakuranetin samples and products after self expression of 8 enzymes. (B) The detection results of (2*S*)-hesperetin and (2*S*)-isosakuranetin standards by LC-MS. The experiment utilized LC-MS from Thermo Electron, Pittsburgh, PA, featuring an electrospray ionization (ESI) source. For the extraction of (2*S*)-hesperetin from the medium, ethyl acetate was employed. The separation and purification of (2*S*)-hesperetin from ethyl acetate were conducted using a BRIX 1860 instrument, which was equipped with a reverse-phase C18 column measuring 25 mm × 70 cm. The mobile phase consisted of 41% (V/V) methyl alcohol, with a flow rate set at 10 mL/min. Detection was carried out at a wavelength of 290 nm, with an injection volume of 5 mL, and the system was maintained at a constant temperature of 25 °C. Following the chromatographic separation, the collected phase underwent vacuum distillation at 40 °C to remove the organic phase. (C) (2*S*)-Hesperetin sample. (D) (2*S*)-Isosakuranetin sample.

**References:**

[1] C. Madzak, B. Tréton, S. Blanchin-Roland. Strong hybrid promoters and integrative expression/secretion vectors for quasi-constitutive expression of heterologous proteins in the yeast *Yarrowia lipolytica*. *J MOL MICROB BIOTECH*, 2 (2000) 207-216, <https://doi.org/10.1007/978-3-642-38583-4_1>.

[2] Y. Mingyu, L. Mengsu, G. Song, R. Xuefeng, Z. Shenghu, R. Yijian, et al. High-Level De Novo Production of (2*S*)-Eriodictyol in *Yarrowia Lipolytica* by Metabolic Pathway and NADPH Regeneration Engineering. *J. Agric. Food Chem*, 72 (2024) 4292-4300, <https://doi.org/10.1021/acs.jafc.3c08861>.
